# Consensus Definition of Blood Samples from the Subcategorized Normal Controls in the Korea Biobank Network

**DOI:** 10.3390/jcm12093080

**Published:** 2023-04-24

**Authors:** Ji Eun Han, Min Kyu Park, Ju Hyun Jin, Jung Ah Lee, Gyeongsin Park, Jong Sook Park, Han-Ik Bae, Seok Joong Yun, An Na Seo, Man-Hoon Han, Hyoungnam Lee, Jae-Pil Jeon, Ji-In Yu, Soon Sun Kim, Jae Youn Cheong

**Affiliations:** 1 Department of Gastroenterology, Ajou University Hospital, Suwon 16499, Republic of Korea; 2 Department of Clinical Pharmacology and Therapeutics, College of Medicine, Chungbuk National University, Cheongju 28644, Republic of Korea; 3 Human Genome Research & Bio-Resource Center, Ajou University Hospital, Suwon 16499, Republic of Korea; 4 The Biobank of Seoul St. Mary’s Hospital, The Catholic University of Korea, Seoul 03382, Republic of Korea; 5 Department of Hospital Pathology, College of Medicine, The Catholic University of Korea, Seoul 06591, Republic of Korea; 6 Department of Allergy & Pulmonology, Soonchunhyang University Bucheon Hospital, Bucheon 14584, Republic of Korea; 7 Department of Pathology, Kyungpook National University Hospital, Daegu 41404, Republic of Korea; 8 Department of Urology, College of Medicine, Chungbuk National University, Cheongju 28644, Republic of Korea; 9 Department of Urology, Chungbuk National University Hospital, Cheongju 28644, Republic of Korea; 10 Division of Biobank, Department of Precision Medicine, Korea National Institute of Health, Cheongju 28159, Republic of Korea

**Keywords:** control definition, healthy control, normal control, biospecimen, biobank, Korea Biobank Network

## Abstract

A control group is defined as a group of people used for comparison. Depending on the type of study, it can be a group of healthy people or a group not exposed to risk factors. It is important to allow researchers to select the appropriate control participants. The Korea Biobank Project-sponsored biobanks are affiliated with the Korea Biobank Network (KBN), for which the National Biobank of Korea plays a central coordinating role among KBN biobanks. KBN organized several working groups to address new challenges and needs in biobanking. The “Normal Healthy Control Working Group” developed standardized criteria for three defined control groups, namely, normal, normal-plus, and disease-specific controls. Based on the consensus on the definition of a normal control, we applied the criteria for normal control participants to retrospective data. The main reason for exclusion from the “Normal-plus” group was blood test results beyond 5% of the reference range, including hypercholesterolemia. Subclassification of samples of normal controls by detailed criteria will help researchers select optimal normal controls for their studies.

## 1. Introduction

In a clinical study, “healthy control” is defined as a person who does not have the disorder or disease being studied but may have other disorders that are not addressed in the specific setting of the research [1]. The term “supernormal” refers to individuals without any evident illness or disorder. Comparing these supernormal individuals with diseased persons often overestimates or measures differences that are irrelevant to clinical practice.

In general, a normal control group is defined as a suitable control group of research participants in an observational study without disease. However, the control group is not necessarily healthy. Most disease-based biobanks collect tissue samples from patients with specific diseases and normal control tissue samples from patients with non-specific diseases or healthy volunteers. Researchers need to establish appropriate standards for research, but the definition of a “normal (or healthy) control” participant is different for each biobank. It is necessary to establish a definition of the normal control group and classify the control samples according to the specified definition to satisfy each researcher’s detailed needs.

Several articles have reported the proper definition of normal control in specific diseases [2,3]; however, none of the references have issued a need for a consensus definition of normal control that can be utilized in the entire disease. There are several examples that justify the need for a consensus definition of normal controls. One example is in the field of medical research, where a control group of individuals with no known health conditions is used as a comparison group to measure the effectiveness of a treatment or intervention being studied. Without a consensus definition of the constituents of a “normal control”, it would be difficult to compare results across different studies and draw valid conclusions. Another example is from the field of psychological research, where a control group of individuals without a specific mental disorder is used to measure the impact of a treatment or intervention for that disorder. Again, without a consensus definition of the constituents of a “normal control”, it would be difficult to compare results across different studies and draw valid conclusions. Finally, in the field of clinical trials, normal controls are needed to compare the results with the experimental group, and without a consensus definition of normal controls, the results of the trial could be inconclusive.

The Korea Biobank Project (KBP) was originally launched in 2008 to establish an efficient nationwide management system to collect, preserve, and utilize human biological samples and their associated data across the country. Since then, the KBP has supported various biobanking activities, including the operational and technical standardization of many university-hospital-based biobanks. These KBP-sponsored biobanks are affiliated with the Korea Biobank Network (KBN), for which the National Biobank of Korea (NBK) plays a central coordinating role for KBN biobanks [http://nih.go.kr/biobank (accessed on 1 March 2023)]. Currently, the KBP sets out for its fourth phase of 5 years (2021–2025) with the vision of biobank-driven R&D innovation in biomedical research and healthcare. To address new challenges and trends in biobanking needs for the 4th phase of KBP, several working groups were organized from the KBN biobanks. Among these working groups, the “Normal Healthy Control Working Group” aimed to provide a guide to normal control collections and definitions in the KBN biobanks. Here, we describe the definition and selection criteria (namely, normal, normal-plus, and disease-specific controls) of normal control groups for KBP.

## 2. Materials and Methods

The “Normal Healthy Control Working Group” was organized by KBN in South Korea. Working group members comprised staff from five university hospitals (Ajou University Hospital, Chungbuk National University Hospital, Catholic University Seoul St. Mary’s Hospital, Soonchunhyang University Bucheon Hospital, and Kyungpook National University Hospital) that belong to KBN.

First, the working group surveyed how “normal (or healthy) controls” are characterized and how each biobank defines a “normal (or healthy) control”. Practitioners from five hospitals belonging to the working group were asked the criteria that had previously been established in each hospital for normal control.

Most institutions collected control samples from participants who had visited a health checkup centers. The criteria of each institution for the definition of healthy controls vary. The three institutions simply defined participants who did not have malignant tumors as healthy controls. Two institutions adjusted more stringent criteria, including normal blood test results, without any specific disease. Candidate participants for blood donation were those who agreed to donate blood during a health checkup. The selection of healthy control participants was done in three steps to confirm that the criteria for normal control were met: (1) A questionnaire including questions regarding the presence of underlying disease, current medication use, and hepatitis B or C virus infection, among others; (2) Interviews were performed with semi-structured questions, including detailed past medical history, current medications, and a willingness to participate by donating a blood or tissue sample; (3) electronic medical records from outpatient clinics were reviewed to obtain epidemiologic information and health checkup data. Two institutions used case report forms to investigate health-related social histories, such as alcohol consumption and smoking history.

The working group members had six conference calls to reach consensus on the definition of normal control. Opinions were gathered through consensus meetings, and conclusions were drawn. A consensus definition of normal control was established by collating committee opinions during six conference calls. The draft consensus statement drawn by the working group members was reviewed and corrected by advisory board members composed of three experts in epidemiology or medical statistics.

## 3. Derivation of Consensus Definition of Blood Samples from the Subcategorized Normal Controls

### 3.1. Establishment of the Consensus on the Definition of Normal Control Participants

Researchers require individualized control groups based on the characteristics of their studies. Therefore, we categorized control subjects into three subtypes (normal-plus, normal, and disease-specific controls). The “Normal-plus” group defines participants who have no demonstrable medical history of disease or disorders recorded in their medical records and health check-ups. The “Normal” group refers to participants without serious illnesses, such as cancer, severe systemic disease, or severe infection; pregnant or lactating women are excluded. The “Disease-specific control” group refers to participants without the disease under study for each specified research topic.

The general exclusion criteria are a history of cancer diagnosis and treatment, the presence of severe systemic disease [cardiovascular (acute myocardial infarction and heart failure), respiratory (severe asthma and emphysema, bronchiectasis), digestive and liver diseases (cirrhosis and ascites, ulcerative colitis, Crohn’s disease), patients receiving dialysis, intractable genetic diseases, and others], severe or active infection, acute infectious diseases (influenza, COVID 19, and pneumonia), human immunodeficiency virus infection, active tuberculosis, and active hepatitis B.

Finally, we reached a consensus on the definition of normal controls. Normal control participants were categorized into three subtypes to select the most appropriate control subjects during individual studies.

**I.**  **Normal**
  - Definition: Participants who do not have a disease or history of clinically significant systemic disease.
  - Requirements:
    (1) No medical history of malignant tumors;
    (2) No clinically significant systemic diseases (cardiovascular, respiratory, gastrointestinal, nervous, urinary, endocrine, musculoskeletal, and mental disorders);
    (3) No medical history of diabetes, hypertension, viral hepatitis, or tuberculosis;
    (4) Exclusion of pregnant or lactating women.
**II.**  **Normal-Plus**
  - Definition: Participants who meet stricter criteria that satisfy the requirements for normal participants and the additional requirements described below.
  - Requirements:
    (1) No excessive alcohol consumption
    (Men: less than 210 g; women: less than 140 g of alcohol per week within 2 years);
    (2) No hazardous cigarette smoking history
    (participants who never smoked or formally smoked more than 15 years ago and have less than a 30-pack-year smoking history);
    (3) No medication is currently being administered for systemic diseases;
    (4) Normal complete blood count and routine serum chemistry;
    (5) No glucosuria or proteinuria documented by urinalysis or a urine stick test;
    (6) No significant symptoms or diseases on the day of sample collection;
    (7) No disease or abnormal conditions detected after reviewing the questionnaire, recording the medical history, and checking vital signs.
**III.**  **Disease-specific control**
  - Definition: Participants who do not have the particular disease being studied.
  - Requirements:
    (1) No medical history of malignant tumors;
    (2) Participants who meet the request of researchers as a disease-specific control;
    (3) Participants who can provide clinical information requested by researchers.

We recommended the acquisition of additional information, including ethnic origin, mental health, stress factors, nutrition, and the parameters (origin/health) of first-degree relatives.

In addition, guidance recommends that data collection and classification should be interview-based. The questionnaire is to be handed out in person to those available for health checkups. The review of electronic medical records to obtain epidemiologic information and medical history for documentation is also recommended, as is querying to that appropriate collectives are filtered.

KBN developed a standard model of KBN disease-based resource clinical information and established an integrated management system to increase the utilization value of human bioresources and the user-customized service system through the smooth operation of the KBN Distribution Support Center. If researchers apply for the blood of subcategorized normal participants, the support center can connect each biobank that has control samples according to the specified definition to satisfy each researcher’s detailed needs.

### 3.2. Application of the Consensus Definition in Retrospective Data

According to the consensus definition of normal controls, we applied the criteria for normal control participants to the data collected between January 2021 and December 2021 at Ajou University Hospital. The amount of alcohol consumed was calculated based on the type of alcoholic beverage, volume, and duration of alcoholic consumption. The quantity of alcohol consumed was expressed in grams of alcohol, which was derived from the following formula:

amount of alcohol consumed (g) = alcohol (%) × volume (mL) × 0.7947 (specific gravity of alcohol).

For the blood test results, we reviewed the white blood cell count, hemoglobin, platelets, hemoglobin A1c, fasting glucose, estimated glomerular filtration rate (eGFR), uric acid, alanine aminotransferase, aspartate aminotransferase, gamma-glutamyl transpeptidase, total cholesterol, and LDL-cholesterol. Urine protein and glucose levels were checked using urinalysis or a urine stick test.

Using the consensus definitions, the retrospective data of 406 control participants were used to assign them to one of the three categories (Table 1). The “Normal-plus” participants comprised the largest portion (214/406, 52.7%) of the target population. The “Normal” participants were 24.6% (100/406) and the “Disease-specific control” participants were 18.7% (76/406). Twelve participants could not be classified because data on their health checkups were unavailable, and four withdrew their informed consent, so their data were not available.

Next, we analyzed the reasons for participants dropping out of the “Normal-plus” group among previously categorized healthy control participants. The main reasons for exclusion from the “Normal-plus” group were results beyond 5% of the reference range of blood tests. The most frequent blood test item for dropout was total cholesterol level (19.4%). Other responsible items were uric acid (4.4%), triglyceride (3.4%), alanine aminotransferase (1.4%), LDL-cholesterol (1.24%), gamma-glutamyl transpeptidase (1.24%), and white blood cell count (1.24%). We also analyzed the data from the last 4 years to check blood test results beyond 5% of the reference range. Table 2 shows similar trends in recent years. A small proportion of reasons for dropout from the “Normal-plus” group were the amount of alcohol consumption and history of current medication.

## 4. Discussion

In experiments, control samples are any type of well-known forensic sample used to ensure that analyses are properly performed and the results are reliable. However, it is not easy to define the “sample from healthy controls” to study various human diseases. A control group is defined as a group of people used for comparison. Depending on the type of study, it can be a group of healthy people (case-control study) or a group not exposed to risk factors (cohort study). For example, participants treated for diabetes might be used as “healthy controls” in cancer biomarker studies. However, participants with diabetes are not “healthy” and could affect the outcome of a cancer biomarker study because diabetes has been known to increase overall cancer risk [4].

From a different point of view, we run into other problems if we limit “healthy” to participants free from disease. The term “supernormal” refers to participants who do not demonstrate any disease or disorder. The comparison of these with diseased patients usually overestimates or measures differences that are not clinically relevant. Choosing stricter or looser criteria might not be ideal when determining the conditions that satisfy control participants.

Population-based biobanks, such as the UK Biobank [5], collect biological samples primarily from healthy volunteers without specific inclusion or exclusion criteria. By contrast, disease-oriented biobanks collect disease-specific biospecimens [6]. They may be focused on a single type of tissue or include biospecimens from different sources that are relevant to diseases, such as cancer [7,8]. It is important to allow researchers to select appropriate control participants. Furthermore, standardization of clinical information is necessary if the clinical and epidemiological information for the selection of control participants differs among institutions. Biobanks belonging to the KBN are disease-oriented biobanks and are most often hospital-based. Each biobank collected samples from healthy volunteers and applied various criteria to define healthy controls. Some biobanks apply strict criteria, whereas others are more flexible in their application. Any definition of a normal control cannot be applied perfectly to all studies using human biospecimens. To solve these problems, we developed standardized criteria for three defined control groups, namely, normal, normal-plus, and disease-specific. Samples were prospectively collected from several biobanks to meet the investigator’s specific requirements [9]. By classifying healthy control participants into three different categories and providing additional information on the samples, researchers can select human biospecimens that satisfy their specific needs.

The consensus definition has several limitations. First, the consensus statement was mostly derived from expert opinion through consensus meetings and not from data-driven evidence because there were few reports on the definition of healthy or normal controls in human samples. Furthermore, we could not find any reports or data on the superiority of specifically defined control participants. Second, most institutions have collected normal control samples from participants who have visited the health checkup center; thus, the samples might be biased. Even though it might not represent the whole population, clinical and laboratory information from the results of health check-ups is usually needed to define the control participants.

## 5. Conclusions

Biobanks play a crucial role in fostering scientific research by guaranteeing quality of biospecimens. The KBN working group developed standardized criteria for the three defined normal control groups. We applied the criteria for normal control participants to real-world data from the KBN biobank and identified that it is possible to systematically obtain a variety of control groups in the biobank network. Subclassification of samples of healthy controls by detailed criteria will help researchers select the most appropriate normal control for their studies.

## Figures and Tables

**Table 1:** Classification of normal control participants according to the consensus definition in retrospective data.

Total (*n* = 406)	*n*	Frequency (%)
Normal	100	24.6
Normal-plus	214	52.7
Disease-specific control	76	18.7
No available data on health check-ups	12	2.95
Withdrawal of informed consent	4	0.98

**Table 2:** The proportion of blood test results beyond 5% as a reason for dropout in the recent 4 years.

Reason for Drop-Out		Total	Drop-Out	Frequency (%)
Blood test results beyond 5%	2022	402	141	35.5
	2021	422	251	59.4
	2020	513	255	49.7
	2019	195	103	52.8

## Data Availability

Not applicable.
